# Wolfram Syndrome: New Mutations, Different Phenotype

**DOI:** 10.1371/journal.pone.0029150

**Published:** 2012-01-04

**Authors:** Concetta Aloi, Alessandro Salina, Lorenzo Pasquali, Francesca Lugani, Katia Perri, Chiara Russo, Ramona Tallone, Gian Marco Ghiggeri, Renata Lorini, Giuseppe d'Annunzio

**Affiliations:** 1 Pediatric Clinic, University of Genoa, IRCCS G. Gaslini Institute, Genoa, Italy; 2 Centro de Investigacion Biomedica en Red (CIBER) de Diabetes y Enfermedades Metabolicas Asociadas (CIBERDEM), Barcelona, Spain; 3 Division of Nephrology, Department of Medicine, Columbia University, New York, New York, United States of America; 4 Laboratory on Pathophysiology of Uremia and Department of Nephrology, IRCCS G. Gaslini Institute, Genoa, Italy; RIKEN Brain Science Institution, Japan

## Abstract

**Background:**

Wolfram Syndrome (WS) is an autosomal recessive neurodegenerative disorder characterized by Diabetes Insipidus, Diabetes Mellitus, Optic Atrophy, and Deafness identified by the acronym “DIDMOAD”. The WS gene, *WFS1*, encodes a transmembrane protein called Wolframin, which recent evidence suggests may serve as a novel endoplasmic reticulum calcium channel in pancreatic β-cells and neurons. WS is a rare disease, with an estimated prevalence of 1/550.000 children, with a carrier frequency of 1/354.

The aim of our study was to determine the genotype of WS patients in order to establish a genotype/phenotype correlation.

**Methodology/Principal Findings:**

We clinically evaluated 9 young patients from 9 unrelated families (6 males, 3 females). Basic criteria for WS clinical diagnosis were coexistence of insulin-treated diabetes mellitus and optic atrophy occurring before 15 years of age. Genetic analysis for *WFS1* was performed by direct sequencing.

Molecular sequencing revealed 5 heterozygous compound and 3 homozygous mutations. All of them were located in exon 8, except one in exon 4. In one proband only an heterozygous mutation (A684V) was found. Two new variants c.2663 C>A and c.1381 A>C were detected.

**Conclusions/Significance:**

Our study increases the spectrum of *WFS1* mutations with two novel variants. The male patient carrying the compound mutation [c.1060_1062delTTC]+[c.2663 C>A] showed the most severe phenotype: diabetes mellitus, optic atrophy (visual acuity 5/10), deafness with deep auditory bilaterally 8000 Hz, diabetes insipidus associated to reduced volume of posterior pituitary and pons. He died in bed at the age of 13 years. The other patient carrying the compound mutation [c.409_424dup16]+[c.1381 A>C] showed a less severe phenotype (DM, OA).

## Introduction

Wolfram Syndrome (WS), also known as DIDMOAD (Diabetes Insipidus, Diabetes Mellitus, Optic Atrophy and Deafness), is an autosomal recessive neurodegenerative disease usually diagnosed in childhood when non autoimmune diabetes occurs with optic atrophy [1], [2]. The condition is very rare with an estimated prevalence of one in 550.000 of children, [3] and with a carrier frequency of one in 354. Mortality is ∼65% before age 35 years, due to central respiratory failure with brainstem atrophy and renal failure secondary to infections [4]. Recently WS was described in a 44 year old patient with diabetes mellitus, central respiratory failure, cognitive impairment, ataxia and parkinsonism [5].

The nuclear gene for WS, *WFS1*, mapped on chromosome 4p16.1 [6], is composed of eight exons: the first is noncoding, the 2^nd^ to the 7^th^ are coding exons, and the 8^th^ is 2.6 kb long spanning 33.4 kb of genomic DNA. The 3.6-kb mRNA encodes an 890-aminoacid hydrophobic and tetrameric protein named Wolframin [7], composed by nine transmembrane segments and large hydrophilic regions at both termini [8]. WFS1 mRNA is expressed in pancreas, brain, heart, skeletal muscle, placenta, lung, liver and kidney. Biochemical studies in cultured cells indicate *WFS1* to be an integral, endoglycosidase H-sensitive membrane glycoprotein that primarily localizes in the endoplasmic reticulum. Evidence suggests that *WFS1* is either a novel endoplasmic reticulum calcium channel or a regulator of channel activity [9], [10].

In 2000 a second locus, *WFS2*, has been mapped on chromosome 4q22-q24 following the linkage analysis of four consanguineous Jordanian families [11]. These patients were not affected by diabetes insipidus, but showed upper gastrointestinal ulceration and bleeding. The ZCD2-encoded protein, ERIS (Endoplasmic Reticulum Intermembrane Small protein), is also shown to localize to the endoplasmic reticulum but does not interact directly with Wolframin.

The aim of our study was to evaluate clinical and molecular observations in nine patients with WS from nine different families and their follow-up in order to establish a genotype/phenotype correlation.

## Materials and Methods

### Etical Statement

The research was conducted according to Declaration of Helsinki and approved by IRCCS Giannina Gaslini Institute Ethical Committee.

### Subjects

We clinically evaluated nine unrelated patients (six males and three females) from nine unrelated families, referred to our Pediatric Clinic. Basic criteria for the Wolfram Syndrome diagnosis were the coexistence of insulin-treated, juvenile-onset Diabetes Mellitus (DM) and Optic Atrophy (OA) occurring before 15 years of age. All analyses were performed after informed written consent obtained from patients or their parents.

### Biochemical and clinical analyses

Autoantibodies against pancreatic β-cells (anti-glutamic acid decarboxylase: GADA, anti-thyrosin phosphatase-like protein: IA-2A, anti-insulin: IAA) were detected by RIA [12], and the analysis of HLA-DQA1 and -DQB1 polymorphisms at genomic levels was performed by polymerase chain reaction/sequence specific primers technique [13]. OA was confirmed by the presence of white papilla with regular and well-demarcated borders at the ophthalmoscope. As regards microangiopathic complications, peripheral neuropathy was assessed by electrophysiological examination, retinopathy was detected by fundoscopy and nephropathy screening was performed by overnight albumin excretion rate. Brain nuclear magnetic resonance (NMR) imaging was performed to assess posterior pituitary and brain structures. Structural renal tract abnormalities were assessed by ultrasonography and intravenous urography. Diagnosis of Diabetes Insipidus (DI) was based on the clinical findings of polyuria, polydipsia, an osmolality of less than 300 mOsm per kilogram of water, or a specific gravity of less than 1010 in a 24 hour urine sample without glycosuria and ketonuria [14]. Hearing impairment was assessed by audiograms across a range of frequencies (125–8000 Hz), and by brainstem auditory evoked potentials to explore the central conduction time of the auditory pathways.

### Molecular analyses

DNA from probands, parents and controls were extracted from whole blood using High Pure PCR Template Preparation Kit (Roche, Mannheim, Germany). Exons and flanking regions of *WFS1* were amplified by PCR using previously described primers [15]. Amplicons were purified with exonuclease I and shrimp alkaline phosphatase (ExoSap-IT, USB Corporation, Staufen, Germany) and then sequenced for both sense and antisense strands using an automated fluorescent sequencing method (Big Dye Terminator Kit v1.1, Applied Biosystems). The products were separated on a ABI PRISM sequencing apparatus 3730 (Applied Biosystems). All variation were validated by sequencing both DNA strand of three independent PCR products.

## Results

Main clinical data of each our patients are reported in Table 1.

**Table 1:** Clinical features and age at diagnosis in nine patients with Wolfram Syndrome.

Patient n°	Sex (M/F)	Current Age(years)	DM(age)	OA(age)	DI(age)	D(age)	Renal tract abnormalities	Neurologic abnormalities
1	M	23	11	10	-	-	-	Anxiety with psychotic hints
2	M	13	4	9 ½	9 ½	9 ½	-	Reduced volume of posterior pituitary
3	M	15	11	14	-	-	-	-
4	F	12	9	9	-	-	-	-
5	M	17	10	7	16	16	-	-
6	F	12	7	11	-	-	double left district	-
7	F	15	14	14	-	10 months	-	Mild cognitive impairment
8	M	12	4	6	7	7	-	Posterior pituitary absence
9	M	17	9	16	-	-	-	-

(Patient n° 2 suddenly died at the age of 13 years).

DM: diabetes mellitus; OA: optic atrophy; DI: diabetes insipidus; D: deafness.

Current age: 2011.

Mutational screening, conducted in our Laboratory, revealed a total of 14 distinct variants (7 missense, 2 nonsense, 5 frameshift) all located in exon 8 except the c. 409_424dup16 in exon 4. Twelve were already described (c.1628T>G, c.2104 G>A, c.1060_1062delTTC, c.1620delGTG, c.2020 G>A, c.1885C>T, c.1230_1233delCTCT, c.1582 T>G, c.2106_2113del8n, c.2051C>T, c.1456 C>T, c.409_424dup16) [15], [16], [17], [15], [18], [19], [20], [21], [21]–[22,23,15,24] and two: the c.2663 C>A, p. S888X; and the c.1381 A>C, p. T461P were novel (Table 2). In order to establish the correct allelic segregation, DNA of parents were sequenced in all cases except in parents of proband 7, whose DNA sample was not available when molecular investigation was performed. None *WFS1* mutations was found in the 100 normal unrelated controls screened by sequencing. None of these mutations has been described in our previous work [25].

**Table 2:** Mutations in the *WFS1* gene (new identified *WFS1* mutations are presented in bold).

Family	Exon	Nucleotide change	Amino acidechange	Type of mutation	Reference
1	8	c.1628T>G;	L543R;	Missense;	Colosimo et al.[2003]
		c.2104 G>A	G702S	missense	Gasparin et al. [2009]
2	8	c.1060_1062delTTC;	F354del;	Frameshift;	Hardy et al. [1999]
		**c.2663 C>A**	**S888X**	**nonsense**	**This study**
3	8	c.1620delGTG;	V540del;	Frameshift;	Colosimo et al.[2003]
		c.2020G>A	G674R	missense	Khanim et al. [2001]
4	8	c.1885C>T;	R629W;	Missense;	Kadayifci et al.[2001]
		c.1230_1233delCTCT	V412fsX440	frameshift;	Giuliano et al. [2005]
5	8	c.1582 T>G	Y528D	Missense	Zalloua et al. [2008]
6	8	c.2106_2113del8nt	F646fs708X	Frameshift	Zalloua et al. [2008]
					Rigoli et al. [2011]
7	8	c.2051 C>T	A684V	Missense	Tessa et al. [2001]
8	8	c.1456 C>T	Q486X	Nonsense	Colosimo et al.[2003]
9	4	c. 409_424dup16	V142fsX251	Frameshift;	Gomez-Zaera et al. [2001]
	8	**c.1381 A>C**	**T461P**	**missense**	**This study**

(Patient 7 carried only one heterozygous mutation).

### Case 1

A male patient, with surgically repaired Tetralogy of Fallot at age of 3 years, received a diagnosis of OA and DM without ketoacidosis at the age of 10 and 11 years respectively. B-cells autoantibodies were absent. Molecular analysis of HLA-DR and HLA-DQ regions revealed 0 heterodimers for type 1 DM susceptibility. He had a sudden anxiety with psychotic hints. The patient carried compound heterozygous mutation: the c.1628T>G, leading to substitution of Leucine with Arginine at codon 543 (L543R), this amino acid change was located in the sixth transmembrane domain of Wolframin. The G702S caused by G>A transition at nucleotide 2104, resulting in the substitution of Glycine with Serine at codon 702.

### Case 2

A male patient with the most severe phenotype, died in bed at the age of 13 years. He showed DM with glycosuria, chetonuria, in absence of ketoacidosis at age of 4 years. B-cells autoantibodies were absent. Molecular analysis of HLA-DR and HLA-DQ regions revealed 0 heterodimers of susceptibility for type 1 DM. Celiac disease was diagnosed at age of 8 years and gluten free diet was prescribed. At age of 9 years he started to suffer from nocturnal apnea. OA (visual acuity 5/10) was diagnosed by fundoscopy at age of 9.5 years. Brain NMR revealed reducing the volume of posterior pituitary and optic nerve atrophy. Also DI and deafness (D) with hearing impairment at the 8000 Hz bilaterally were diagnosed at the age of 9.5 years. He harbored in-frame 3-bp deletion, nucleotides 1060_1062, in exon 8, that results in the loss of Phenylalanine 354 residue. According to the predicted structure of the Wolframin protein [17] residues 350 and 354 are likely to be located in the first transmembrane domain. However, the ultimate effect of these deletions cannot be anticipated yet. He also carried a novel nonsense mutation in exon 8 c.2663 C>A leading to S888X, causing a premature stop codon at residue 888 which removes 2 high conserved aminoacids from the carboxy tail.

### Case 3

A male patient developed insulin treated DM without ketoacidosis at 11 years of age. B-cells autoantibodies were absent. Molecular analysis of HLA-DR and HLA-DQ regions revealed 0 heterodimers for type 1 DM susceptibility. OA was diagnosed at the age of 14 years. DI and D were absent up to now. He harbored an in-frame deletion, 1620delGTG, causing the loss of one Valine residue (V540del) and a missense mutation G674R resulting in the substitution of Glycine with Arginine at codon 674.

### Case 4

A female patient developed insulin-treated DM and OA at the age of 9 years. B-cells autoantibodies were absent. Molecular analysis of HLA-DR and HLA-DQ regions revealed 0 heterodimers of susceptibility for type 1 DM. DI and D were not present up to now. She was found to be compound heterozygous: the R629W that converted Arginine in Tryptophan at codon 629 and the in-frame deletion of four nucleotides at position 1230 resulting in a premature stop codon, predicting a truncated protein of 450 aminoacids (V412fsX440).

### Case 5

A male patient presented at the age of 7 years impaired vision, mild papillary pallor, presence some microaneurysms at fundoscopy, diffuse reduction of sensitivity: genetic testing for Leber's optic atrophy was positive. He suffered from insulin-treated DM with glycosuria, chetonuria, ketoacidosis since the age of 10 years. B-cells autoantibodies were absent. Molecular analysis of HLA-DR and HLA-DQ regions revealed 1 heterodimer for type 1 DM susceptibility. At the age of 16 years D with loss of sensorineural hearing for high tones, confirmed by audiometric test, and DI were diagnosed. He carried a homozygous missense mutation, inherited from both his parents, c.1582 T>G resulting in the substitution of Tyrosine with Aspartic Acid at codon 528 (Y528D).

### Case 6

A female patient developed insulin treated DM at 7 years of age. B-cells autoantibodies were absent. Molecular analysis of HLA-DR and HLA-DQ regions revealed 2 heterodimers for type 1 DM susceptibility. At the age of 11, OA was diagnosed by fundoscopy; brain NMR signs of optic nerve impairment. DI, D were absent up to now. Urinary tract ultrasonography showed a double left district. She was found to have a homozygous 8-bp deletion from both alleles, nucleotides 2106_2113 in exon 8, resulting in a frameshift at codon 646 (Phenylalanine) and an early STOP codon, predicting a truncated protein of 708 amino acids missing part of the transmembrane domains and its hydrophilic carboxy tail.

### Case 7

A female patient had profound deafness from the age of 10 months confirmed by audiometric test; at age of 5 years a cochlear implant was inserted. She showed insulin-treated DM, with glycosuria, chetonuria, ketoacidosis, and OA at 14 years of age. B-cells autoantibodies were absent. Molecular analysis of HLA-DR and HLA-DQ regions revealed 0 heterodimers of susceptibility for type 1 DM. DI is absent up to now. She showed mild cognitive impairment. Only one heterozygous mutation was found: the A684V caused by C>T transition at nucleotide 2051 resulting in the substitution of Alanine with Valine at codon 684. She also carried 10 polymorphisms (rs1801213 CG Het, rs1801212 AG Het, rs1801206 CT Het [V395V associated with DM1[26]], rs1801208 GA Het [R456H, associated with risk of Type 2 Diabetes [27], suicide [28] non autoimmune Diabetes [29]] rs1801214 CT Het, rs1046314 AG Het and rs1046316 AG Het [K811K and S855S, associated with DM1 [26]], rs1046317 CT Het, rs1046319 CT Het, rs1802453 GA Het). The A684V could not be confirmed in her parents since no DNA sample from their was available at the time of the screening of *WFS1* gene.

### Case 8

A male patient showed all the clinical features of the syndrome: he suffered from insulin-treated DM since the age of 4. B-cells autoantibodies were absent. Molecular analysis of HLA-DR and HLA-DQ regions revealed 0 heterodimers for type 1 DM susceptibility. At the age of 6 years, OA was found and at age of 7 years, bilateral sensoneurial deafness and DI were diagnosed. Brain NMR revealed reducing the thickness of the nerve and chiasm and optic tracts, a nodule of heterotopic gray matter in the left anterior frontal periventricular. The posterior pituitary was not present. He resulted to be homozygous for the nonsense mutation 1456 C>T leading to a premature stop codon at residue 468 (Q486X), inherited from both his parents. This variant is predicted to give rise to an abnormal protein, missing part of the transmembrane domains and its hydrophilic carboxy tail.

### Case 9

A male patient suffered from insulin-treated DM since the age of 9 years. Autoantibodies against β-cells were absent. Molecular analysis of HLA-DR and HLA-DQ regions revealed 0 heterodimers for type 1 DM susceptibility. At the age of 16 years OA was diagnosed. DI and D were absent up to now. He carried a compound heterozygous mutation: the 409_424dup16 in exon 4 is predicted to produce an aberrant protein; assuming that no splicing alterations occur, translation will follow until residue 251, where a stop codon is created [24]. The other identified change was a novel missense mutation c.1381 A>C resulting in the substitution of Treonine with Proline at codon 461.

## Discussion

We report clinical and molecular observations about nine unrelated patients with WS and their follow-up. Up to now a wide spectrum of *WFS1* mutations were described [22]. In our study 14 different molecular defects were found. Thirteen were identified in both alleles: five probands carried compound heterozygous for *WFS1* mutations; three had *WFS1* defects in homozygous state. In one proband (patient 7) only an heterozygous mutation (A684V) was detected. Two novel mutations the S888X, and the T461P were found in patients 2 and 9 respectively (Table 2). All cases exhibited the main clinical features of WS.

The median age of the patients at the time of DNA analysis was 15,1 years (range 12–23).

Diabetes Mellitus is usually the first symptom occurring before age of 10 years, with optic atrophy at a median age of 11 years [30]. In our series, the average age at onset of diabetes was 8,7 years (range 4–14). WS-associated DM shows clear differences respect to autoimmune type 1 diabetes. WS-associated DM is due to loss of pancreatic β-cell function and number, as confirmed by autoptic studies, without markers for an autoimmune process [31].

Optic Atrophy occurs by definition in all patients with WS and is progressive. All our patients had OA and its identification was concomitant or after diabetes diagnosis except in case n° 1 and 5: where OA occurred before DM diagnosis. OA was diagnosed and confirmed by brain NMR in the first decade of life in 5 cases and between 11 and 16 years of age in the other 4. Although our patients are genetically heterogeneous, ophthalmological findings and age at detection are similar to other series [4], [32], [33]. The pathogenesis of OA could result from the effects of WS mutation on the survival of retinal ganglion cells, and in that case the mutation would lead to anterograde atrophy of retinal axons and shrinkage of the optic nerve. Retinal ganglion cells and optic nerve glial cells were found to be strongly labelled, suggesting that dual dysfunction of Wolframin in these cells might explain the progressive optic nerve atrophy reported in WS [34].

The development of polyuria and/or enuresis can indicate diabetes insipidus, which usually appears during the second decade. In our series only three patients showed DI at younger age (n° 2, 5, 8 at age of 9_1/2_, 16, 7 years respectively). (Table 1).

Sensorineural Deafness, confirmed by audiograms, was diagnosed in three patients at a mean age of 10.8 years (range 7–9_1/2_). Proband 7 had profound deafness from infancy requiring a cochlear implant. Such early onset of severe deafness has not been usually reported in Wolfram Syndrome. Hearing impairment reported in 66% of WS patients, ranging from congenital deafness to mild, progressive hearing impairment. Other case series reported deafness at a median age of 16 years in 60% of cases [35]. Audiometric features include a severe auditory threshold shift, more evident for the medium/high frequencies. Auditory impairment could be a consequence not only of a dysfunction of cochlear neurons and VII nerve fibers, but also of the central nervous pathways in brainstem and inferior colliculus [36].

Involvement of urinary tract represents another serious complication in WS patients and is estimated to occur in up to 90% of patients, being diagnosed during adolescence [37] or adulthood [38]. In our cases has been found only one patient (n° 6) with renal tract abnormalities: the ultrasonography of the urinary tract showed a double left district.

Neuropsychiatric manifestations are reported in the third decade. However Chaussenot A et al, who showed that the onset of neurologic symptoms, with a median age of 15 years, is much earlier than previously reported (mean age 30 years) [39]. In the majority of our patients behavioral problems or depression were evident at much younger ages. This could be due to the fact that previous studies focused on cerebellar ataxia and brainstem involvement as main neurological involvement in WS.

In many cases, it can be difficult to tease out the effect of multiple disabilities and reactive depression from a true depressive illness. It is important to involve a pediatric psychiatrist as part of the multidisciplinary team, particularly if there is a seminal event such as a first seizure. Such events can have devastating psychological consequences for an adolescent.

Wolframin *in vivo* is organized as a tetramer which originates a membrane Ca^2+^ channel of the Endoplasmic Reticulum (ER) and lack of function of *WFS1* determines apoptotic input signaling [40]. The ER has many roles, which include post-translational modification, folding and assembly of newly synthesized proteins such as insulin. Perturbations in ER function cause an imbalance between these processes, leading to accumulation of misfolded and unfolded proteins in +the organelle, a state called ER stress [41].

**Figure 1:**
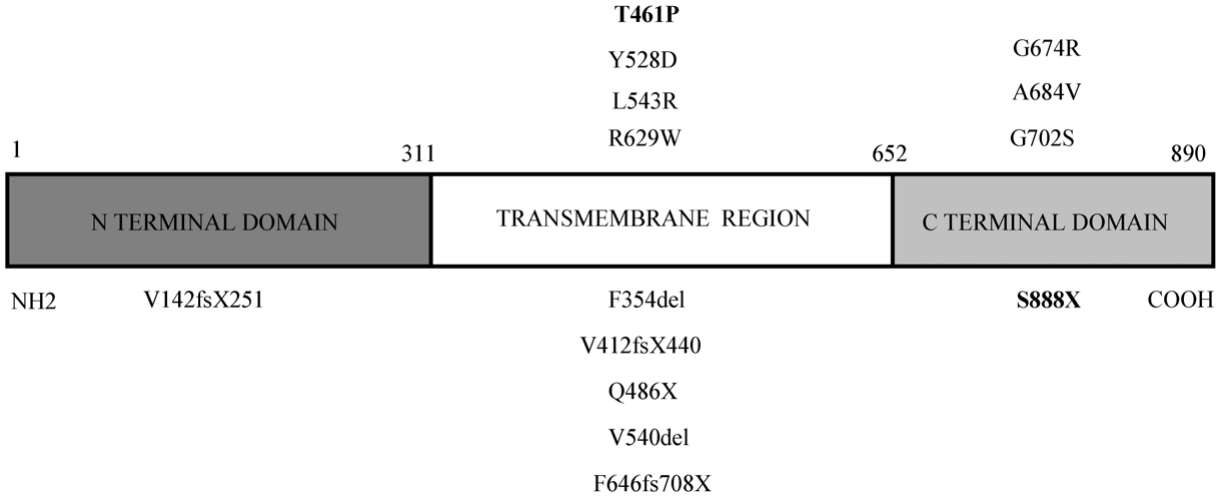
Schematic representation of Wolframin showing all our identified mutations. The novel are in boldface.

Except the V142fsX251 found in exon 4, all the detected mutations are located in exon 8, corresponding to the transmembrane region and carboxytail of Wolframin protein. The majority of mutations described in our study were localized to the predicted transmembrane domains (64,3%, n = 9/14). Mutations in the amino-terminal domain were identified on 1 (7,1%) and in the carboxy-tail on 4 (28,6%) respectively (Fig. 1). This is in agreement with other studies in Italian and world-wide populations [15]. All causative mutations were identified on both alleles except in one case in which only one heterozygous mutation was found.

The predicting information that genetic analysis can give regards the difference between inactivating and non-inactivating mutations. The age at onset of DM was chosen as an indicator of disease severity. Missense mutations and 3 bp deletions, resulting in a deletion of one amino acid, were considered non-inactivating mutations. Nonsense, frameshift mutations, deletions and insertions of more than 3 bp were considered inactivating mutations [42]. Among the five patients with *WFS1* compound heterozygous mutation, two carried 2 non-inactivating mutations, three carried 1 inactivating and 1 non-inactivating. Their ages at diabetes clinical onset were 11 years and 7,3 years, respectively.

Among the three patients with *WFS1* homozygous mutation two carried inactivating and one non-activating; their mean ages at onset of diabetes were 5,5 and 10 years respectively.

Patient 7 carried only one non-inactivating mutation and the age at onset of diabetes was 14 years.

However, the only patient harbouring one mutations outside exon 8 showed a mild phenotype: the onset of diabetes mellitus and optic atrophy was 9 and 16 years respectively. A hearing deficit, diabetes insipidus, renal tract and neurologic abnormalities were absent. We could observe in our study that inactivating mutation give a more severe phenotype that non inactivating mutation, although clinical symptoms are different, according to a previous observation [9].

In our study, seven cases among 9 cases were identified mutations already published elsewhere. To clarify the actual genotype/phenotype correlation, we compared clinical manifestaions of the subjects sharing the same mutations. The clinical features caused by the missense mutation Y528D and the delection F646fs708X, both found in homozygous state in proband P5 and in P6, respectively; were similar to what reported by Zalluoa 2008 and Zalluoa 2008-Rigoli 2011. Unfortunately, the combination of compound heterozygous defects found in our series has never been reported in literature. So, it is difficult to compare the phenotype these our patients with what reported in other studies. However, validation of any genotype–phenotype correlation must await detailed functional analysis of mutations on a cellular and molecular level.

If the mutation causing the syndrome in a family is known, it is possible to offer genetic counselling and prenatal diagnosis. Prenatal diagnosis is possible analyzing DNA extracted from fetal cells by amniocentesis at approximately 15–18 weeks gestation or chorionic villus sampling at approximately 10–12 weeks gestation. [43], [44].

WS is a progressive neurodegenerative disorder, that usually appears in adolescence and requires careful endocrinological evaluation aimed to start an early and adequate hormonal substitutive therapy. Even if WS is characterized by a wide differential diagnosis including other causes of neurodegeneration, it should be suspected in a young with diabetes mellitus and optic atrophy, hearing loss, polyuria and polydipsia but adequate glucose control [44]. Genetic test represents the best opportunity to confirm the clinical diagnosis and to propose prenatal diagnosis.

In conclusion, we have analyzed the *WFS1* in nine WS patients. Our study increases the spectrum of *WFS1* mutations with two novel variants.
